# Development of a polyvinyl alcohol/sodium alginate hydrogel-based scaffold incorporating bFGF-encapsulated microspheres for accelerated wound healing

**DOI:** 10.1038/s41598-020-64480-9

**Published:** 2020-04-30

**Authors:** Maedeh Bahadoran, Amir Shamloo, Yeganeh Dorri Nokoorani

**Affiliations:** Department of Mechanical Engineering, Sharif University of Technology, Tehran, Iran

**Keywords:** Tissue engineering, Biomedical engineering

## Abstract

In the present study, a hybrid microsphere/hydrogel system, consisting of polyvinyl alcohol (PVA)/sodium alginate (SA) hydrogel incorporating PCL microspheres is introduced as a skin scaffold to accelerate wound healing. The hydrogel substrate was developed using the freeze-thawing method, and the proportion of the involved polymers in its structure was optimized based on the *in-vitro* assessments. The bFGF-encapsulated PCL microspheres were also fabricated utilizing the double-emulsion solvent evaporation technique. The achieved freeze-dried hybrid system was then characterized by *in-vitro* and *in-vivo* experiments. The results obtained from the optimization of the hydrogel showed that increasing the concentration of SA resulted in a more porous structure, and higher swelling ability, elasticity and degradation rate, but decreased the maximum strength and elongation at break. The embedding of PCL microspheres into the optimized hydrogel structure provided sustained and burst-free release kinetics of bFGF. Besides, the addition of drug-loaded microspheres led to no significant change in the degradation mechanism of the hydrogel substrate; however, it reduced its mechanical strength. Furthermore, the MTT assay represented no cytotoxic effect for the hybrid system. The *in-vivo* studies on a burn-wound rat model, including the evaluation of the wound closure mechanism, and histological analyses indicated that the fabricated scaffold efficiently contributed to promoting cell-induced tissue regeneration and burn-wound healing.

## Introduction

Skin, the largest organ of the body, serves as the first protective barrier against the external environment; hence, it is constantly exposed to wounds and burn injuries^1^. For the wounds limited to the epidermis, or superficial parts of the dermis, the self-healing ability of the skin is sufficient to repair the damaged tissue^2,3^. However, the wounds that extend entirely across the dermis or reach the hypodermis, such as deep ulcers or full-thickness burns, skin grafting would be necessary^2,3^. The poor availability of the healthy tissues as autografts and the severe risks of using non-self-tissues as allografts have made tissue-engineered skin substitutes a more suitable and permanent solution for tissue regeneration^1,4,5^. Despite the significant attempts to develop the skin substitutes over the last decades, the attainment of an ideal skin scaffold has remained a challenging issue due to the complexity associated with the wound healing process.

Recent advances in skin tissue engineering, have directed the researchers towards developing the bioactive scaffolds capable of sustained and controlled drug delivery to the wound site^6,7^. Unlike traditional wound dressings such as gauze and cotton wool, these scaffolds are designed for promoting the biological processes of wound healing^7^. Besides, they can be used as biocompatible carriers for a wide range of medications that are involved in the wound healing process, either directly for removing necrotic tissues or indirectly for releasing the growth factors and antimicrobial drugs to support tissue regeneration^7^. The bioactive scaffolds can be made of natural polymers, such as sodium alginate (SA) and gelatin, as well as synthetic ones, for instance, polyvinyl alcohol (PVA) and polycaprolactone (PCL), or a combination of them^4,7–9^.

The utilization of growth factors (GFs), as the regulators of cellular behaviors, is a promising approach to accelerate the healing process and cell-induced tissue development^6,10,11^. Among various GFs, basic fibroblast growth factor (bFGF) is highly efficient in skin regeneration, via promotion of fibroblasts migration and proliferation, and stimulating these cells to produce collagenase^12,13^. In addition, bFGF is a potent angiogenic factor, which stimulates the proliferation of capillary endothelial cells^12^. Nevertheless, the use of bFGF possesses limitations due to its short half-life and rapid diffusion^14,15^. In fact, the GFs, whether produced physiologically, injected, or applied topically are degraded by active proteases at the wound site, leading to impaired wound healing^6,16^.

In order to overcome the problems of using growth factors, a sustained drug delivery system (DDS) is required to preserve GF bioactivity over the healing process, by following a controlled release profile. Accordingly, multiple DDSs have been developed, mainly focusing on polymer micro-^17^ and nanospheres^18^, liposomes^19^, nanofibers^20^, as well as hydrogels^21^, and combinations of the afore-mentioned techniques, including drug-loaded microspheres encapsulated within hydrogels^22^ or nanofibers embedded within hydrogels^23^. Among these methods, the encapsulation of GFs in microspheres incorporated into hydrogel structures is highly appreciated, as it results in a more controlled release kinetics compared to the release profile from either hydrogels or microspheres^22,24–26^. In this hybrid system, the microspheres are mainly responsible for controlling drug release, while the hydrogel is primarily a scaffold to retain cells within its structure^27^. Given the high biocompatibility of the natural polymers and time tunable characteristics of the synthetic ones, the simultaneous use of these polymers in fabricating hydrogels leads to the development of an optimized substrate, which is capable of efficiently mimicing extra-cellular matrix (ECM) of the skin tissue^4,28^.

The goal of the present study is to develop a novel hybrid system including PCL microspheres as the biocompatible bFGF carriers, embedded into a hydrogel substrate to accelerate the wound healing process. An optimized combination of PVA and SA has been utilized to create the hydrogels in this study.

PVA, which is a biocompatible polymer, has been prevalently utilized in advanced biomedical applications, such as wound dressings^29^. This polymer represents desirable properties, including non-toxicity, high hydrophilicity, excellent processability, chemical resistance and high mechanical strength^30,31^. However, PVA hydrogels may form undesirable elastic and stiff membranes, which restricts their use as wound dressings^32^. SA, as a blended material, has been utilized to modify the clinical properties of PVA hydrogels for wound dressing applications^30,33^. This polymer presents high protein absorption ability, fast biodegradability, high hydrophilicity, antibacterial activity, and pleasant hemostatic and biological properties^4,33^. As a result, PVA/SA hydrogels benefit from the desirable features of both polymers; PVA provides sufficient mechanical properties, whereas SA improves the physical and biological characteristics of the samples. PCL is an FDA-approved polymer which is extensively used for constructing controlled drug delivery systems, warranting a long-term release due to its markedly slow degradation rate, which prevents a burst release^22,34,35^.

The main novelty of the present study relates to the fabrication a hybrid drug delivery system providing a sustained and controlled bFGF release kinetics, which can be easily applied at the wound site due to the hydrated and smooth hydrogel substrate. Besides, the proposed scaffold offers a stable structure with favorable mechanical properties due to the presence of PVA and benefits from sufficient physical and biological properties of SA. In the present study, the first phase includes the independent optimization of the hydrogel substrates by using three different proportions of the blended polymers. In the second phase, the hybrid system has been created by adding the microspheres into the optimal scaffold obtained from the first phase, followed by assessing its characteristics *in-vitro* and *in-vivo*.

## Materials and methods

### Materials

Polycaprolactone (PCL) (Mw = 80 kDa), sodium alginate (SA) and bovine serum albumin (BSA) were purchased from Sigma Aldrich (USA). Polyvinyl alcohol (PVA) (Mw = 72 kDa), chloroform, and bFGF were purchased from Merck (Germany). All products were used as received.

### Preparation of PCL microspheres

PCL microspheres in which bFGF, and BSA as a model protein, are encapsulated, were prepared by using the double-emulsion solvent evaporation technique, as explained in ref. ^36^. Briefly, BSA (1% w/v) was dissolved in phosphate buffer saline (PBS, pH 7.4) containing PVA (1% w/v) to obtain the internal aqueous phase (W_1_). 0.1 ml of W_1_ was then poured into 1 ml of PCL solution in chloroform (3% w/v), followed by emulsification for 2 min at 12700 rpm using a homogenizer (MTOPS SR30, South Korea). The primary emulsion (W_1_/O) was subsequently added by dropwise into 25 ml of 5% (w/v) PVA/PBS solution and stirred at 700 rpm for 5 min to obtain the double emulsion (W_1_/O/W_2_). For complete evaporation of the organic solvent, which results in microspheres hardening, the mixture was magnetically stirred for another 3 h. Solid microspheres were eventually collected through centrifuging, followed by filtering and washing by distilled water.

The bFGF-loaded microspheres, utilized in cell viability and antibacterial assessments, were prepared using the same method, but instead of BSA, 4 μgr of bFGF was added into 0.1 ml of the internal aqueous solution. The selected concentrations of the polymers to fabricate the microspheres were selected based on the previous results from the evaluation and optimization of PCL microspheres in refs. ^22,34^.

### Preparation of hydrogels and hybrid (microsphere/hydrogel) system

PVA was dissolved in distilled water under magnetic stirring at 80 °C to prepare a 10% (w/v) solution and was cooled down to the room temperature. SA solution in distilled water was also prepared at a concentration of 3% (w/v) at 40 °C. Subsequently, different proportions of PVA and SA (SA = 10, 30, and 50% v/v of the SA/PVA combinations) solutions were blended with a magnetic stirrer for 30 min. The homogenous gel precursors were then poured in Petri dishes, followed by freezing at −20 °C for 20 h and thawing at 25 °C for 4 h for three consecutive cycles. The hydrogels were eventually freeze-dried for 24 h for water sublimation and increment of structural porosity. The approximate thickness of the final scaffolds was around 3 mm.

To fabricate the hybrid system, pre-made PCL microspheres were initially dispersed into 5 ml of the gel precursor. The mixture was then magnetically stirred to become completely homogeneous. Afterward, the freeze-thawing and freeze-drying processes were performed, as mentioned above.

### *In vitro* characterization of the samples

#### Scanning electron microscopy (SEM) analysis

The morphology of the microspheres, hydrogels with/without microspheres, and cell-seeded hydrogel were analyzed using scanning electron microscopy (SEM, XL30 model, Philips). For this purpose, dry samples were sputter-coated with gold to a thickness of 200–500 Å and positioned on a metal stub for observation under SEM.

To evaluate the cell adhesion on hydrogels via SEM images, mouse connective tissue fibroblast cells (L929 cells), purchased from Pasteur Institute of Iran, were seeded at a density of 5 × 10^4^ cell/well on sterilized hydrogel samples, placed in a 24-well plate. After incubating at 37 °C for 5 days, samples were fixed by immersing them in a 2.5% glutaraldehyde (GA) solution for 2 h. GA was subsequently removed, and hydrogel samples were washed using PBS. The cell-seeded hydrogels were then dehydrated by increasing ethanol concentration starting from 50% to 100% with a step of 10%, every 10 min.

SEM Images analyses for measuring the pore size and the porosity percentage of the scaffolds were performed using ImageJ® software. The values are reported as the mean ± standard deviation.

#### Swelling ratio

To investigate the water absorption ability of the samples, their weights were initially measured immediately after freeze-drying (W_d_). The specimens were then soaked in PBS at room temperature. In the next step, their wet weights (W_w_) were measured at specified time intervals, after the removal of excess water from the surface of the samples with the help of filter papers. The swelling ratio (SR) was calculated according to the following formulation^37^. The values are reported as the mean ± standard deviation (n = 4).

1 $$ \% \,Swelling\,ratio=\left(\frac{Ww-Wd}{Wd}\right)\times 100$$

#### Mechanical tensile properties

The freeze-dried hydrogels were initially immersed in PBS for 1 h, to present their realistic gel-like behavior. Afterward, the mechanical properties of the hydrogel samples with a rectangular shape was characterized using a universal tensile testing machine (H10KS, Hansfield) with a constant extension rate of 2.5 mm/min. The values are reported as the mean ± standard deviation (n = 3).

#### *In-vitro* degradation

To study the *in-vitro* degradation rate of the samples, the enzymatic degradation method was used to simulate the *in-vivo* condition of skin wounds^38,39^. For this purpose, freeze-dried hydrogels were suspended in PBS (pH = 7.4) containing 0.1 mg/ml lysozyme enzyme (Sigma-Aldrich, USA) after measuring their initial weight (W_i_). They were subsequently placed in a shaker incubator at 50 rpm at 37 °C. The buffer was replaced every two days. At specific time points, each sample was washed by distilled water to remove the remaining salts. They were then freeze-dried for 24 h, and the final weights of the dry specimens (W_f_) were measured. Degradation percentage was calculated using the following equation. The values are reported as the mean ± standard deviation (n = 4).

2 $$ \% \,Degradation=\left(\frac{Wi-W{\rm{f}}}{Wi}\,\right)\times 100$$

#### *In-vitro* BSA and bFGF release

In order to evaluate the release kinetics of the drug from the hydrogels, microspheres alone and microspheres incorporated into the hydrogels, the *in-vitro* drug release was studied using BSA, as a model protein in the first step. To this end, the hydrogel samples, soaked in PBS solution (pH 7.4), were shaken at 50 rpm at 37 °C. The obtained microspheres were also suspended in PBS solution (pH 7.4), and maintained in a shaker incubator at 50 rpm in 37 °C.

The absorption spectra of BSA at different concentrations (Fig. S1(A) in the Appendix), obtained using a spectrophotometer (Hach, DR 5000), indicated the particular absorption peak at 280 nm, as reported previously in ref. ^40^. Accordingly, at each time interval, the concentration of BSA released in PBS was determined by measuring the solution absorbance at 280 nm via spectrophotometer and based on the calibration curve shown in Fig. S1(B) in the Appendix. The buffer solution was also replaced at each time point after measurement. The values are reported as the mean ± standard deviation (n = 5).

Due to the difference between BSA and bFGF in terms of their molecular weights and isoelectric points, a similar procedure was utilized to study the release kinetics of bFGF loaded microspheres incorporated in hydrogel substrates, but by using a NanoDrop 2000/2000c Spectrophotometer (n = 3). Besides, in order to investigate the release kinetics of BSA and bFGF loaded specimens, the Peppas-Ritger equation was used to fit the data according to the following equation^41^.

3 $$\frac{{M}_{t}}{{M}_{\infty }}=k\times {t}^{n}$$

In equation (3), M_t_/M ∞, t, and n represent the fractional solute release, the release time and the diffusional exponent characteristic of the release mechanism, respectively. In addition, k is a constant.

#### BSA encapsulation efficiency

The extraction of BSA from PCL microspheres was studied to determine the encapsulation efficiency (EE) of the drug carriers based on the method used in^22,34,42^. Briefly, the microspheres were initially dissolved in 3 ml of chloroform under magnetic stirring. The obtained solution was added to 3 ml of PBS (pH 7.4) and then stirred overnight. Afterward, the mixture was centrifuged at 13000 rpm for 10 min using microcentrifuge (Hettich, Germany) to extract the aqueous phase containing BSA. BSA concentration was subsequently quantified by measuring the absorbance of the solution at 280 nm using a spectrophotometer. The EE of microspheres was calculated according to the following formulation. The values are reported as the mean ± standard deviation (n = 4).

4 $$ \% \,EE=\left(\frac{Measured\,BSA\,content}{Teorithical\,BSA\,content}\right)\times 100$$

#### Water vapor transmission rate

In the present study, the water vapor transmission rate (WVTR) of the hydrogel substrates was measured using the method mentioned in ref. ^43^. Briefly, the hydrogel samples were put at the mouth of bottles with a diameter equal to 15 mm, which contained 10 ml of distilled water. The bottles were initially weighed (W_0_) and then placed into an incubator at 37 °C for 24 h. Then, the weights of the containers were measured (W_1_) and the WVTR was calculated according to the following equation. In this equation, A represents the area of the mouth of the bottle in m^2^. The values are reported as the mean ± standard deviation (n = 3).

5 $$WVTR=\frac{({W}_{0}-{W}_{1})}{A\times 24}g/{m}^{2}h$$

#### Antibacterial performance evaluation

To evaluate the antibacterial performance of the prepared scaffolds, the antibacterial inhibition zone was investigated against Staphylococcus aureus (abbreviated as S. aureus, Gram-positive) and Escherichia coli (abbreviated as E. coli, Gram-negative), as the main species responsible for wounds infections^44–46^. To this end, the sterilized nutrient broth was initially inoculated with bacterial strains, followed by being incubated at 150 rpm for 24 h at 37 °C. The solution was then diluted to obtain the optical density (OD) of 1 at 600 nm using a spectrophotometer. Subsequently, 20 ml of the sterilized agar was poured into each 100-mm diameter petri dish and gelled in a short time, on which the 0.1 ml of the diluted bacterial solution was poured. The circular samples with a diameter of 17 mm were placed on the solid agar and incubated at 37 °C. After 24 h, antibacterial inhibition zones were measured. The values are reported as the mean ± standard deviation (n = 3). Besides, in order to investigate the effect of pure PVA on the S. aureus activity, a similar procedure of antibacterial assay was performed, and its result is shown in Fig. S2 in the Appendix.

#### *In-vitro* cell adhesion and proliferation

In this study, fourth passage mouse connective tissue fibroblast cells (L929 cells), purchased from Pasteur Institute of Iran, were used for MTT (3-(4,5-dimethylthiazol-2-yl)-2,5-diphenyltetrazolium bromide) cell viability assay, which was performed based on the international standard ISO 10993-5:2009^47^. In this regard, hydrogels were initially submerged in 70% ethanol for 24 h in 24-well plates. Then, Each side of the samples was exposed to ultra-violet radiation for 1 h. After sterilization, hydrogels were washed 3 times for 15 min in sterilized PBS to remove the remaining ethanol from the specimens. To prepare the samples for cell culture, they were submerged in DMEM (Dulbecco’s modification of Eagle medium, Gibco) and incubated at 37 °C in a humidified atmosphere containing 5% CO_2_ (cell culture incubator). The cell culture medium was removed after 24 h incubation and cells were seeded on the hydrogels at a density of 5 × 10^4^ cells/ well. Polystyrene tissue culture plate (TCP) was also seeded with the same density of cells as the control group. Afterward, the complete cell culture medium containing 89% DMEM, 10% fetal bovine serum (FBS, Gibco), and 1% penicillin/streptomycin (Gibco) was added into the wells. The plates were placed in cell culture incubator. The culture medium was also replaced every 2 days. To perform MTT assay on the 1st, 3rd, and 5th days of the experiment, 550 *μl* of the culture medium of each well was replaced with 50 *μl* of MTT solution (Sigma, 5 mg/ml). After incubating the plates containing MTT solution at 37 °C for 3 h, the culture medium was removed from the wells, and 500 *μl* of dimethyl sulfoxide (DMSO, Gibco) was added to the wells to dissolve the blue-violet insoluble formazan in living cells. The plates were then incubated at 150 rpm at 37 °C for 20 min. Finally, cell proliferation was evaluated by measuring the optical density of the final solution in each well using an Elisa plate reader at 570 nm. The values are reported as the mean ± standard deviation (n = 4).

### *In-vivo* assay

All *in-vivo* studies were performed considering institutional ethical protocols approved by Sharif University of Technology research committee. These guidelines include appropriate methods for feeding, locating, and sacrificing rats in the *in-vivo* experiments. In each group, the number of rats was initially equal to n = 5.

#### Wound closure mechanism

All animal experiments were carried out based on the “Guide for the care and use of laboratory animals”. As a burn-wound animal model, male Wrister rats (8 weeks old, 200–250 g) were selected. After anesthetizing the rats via injection of xylazine (5 mg/kg body weight) and ketamine hydrochloride (100 mg/kg body weight), their dorsal hair was shaved. The wound site was decontaminated, employing 10% povidone-iodine. Then, the electric device heated at 85 °C contacted the dorsal skin of the rats for 10 sec. After the burn-wound area was cooled down, the Wrister rats were randomly divided into four groups. The first group was treated with the sterilized hybrid scaffold, consisting of bFGF-encapsulated microspheres embedded into the optimized hydrogel (H-bFGF), which was selected based on the *in-vitro* studies. The rats in the second group were treated with the drug-free optimized hydrogel structure (O-H). A commercial gel (Comfeel Plus, Coloplast), as the positive control (PC), was topically applied to the third group. Finally, the burn-wounds of the fourth group were covered with a sterile layer of gauze, as the negative control (NC). All of the applied treatments were fixed with sterile gauze and medical fixation tape. The scaffolds, as well as the control dressings, were replaced by new ones every week. On the 7th, 14th, and 21st days post-burn, the dressings were removed to assess the wounds and analyze the percentage of wound closure. The wound reduction percentage was calculated with the following equation^48^:

6 $$ \% \,Wound\,Closure=\left(\frac{A0-At}{A0}\right)\times 100$$

Where A_0_ and A_t_ represent the initial wound area and the area after each time interval, respectively, calculated using the ImageJ® software. The values are reported as the mean ± standard deviation (n > = 4).

#### Histological/histomorphometric study

The skin tissues containing wound sites were excised and immediately fixed in 10% neutral buffered formalin (PH. 7.26) for 48 h. After embedding in paraffin, the biopsies were sectioned to a 5 *μ*m thickness. Finally, the sections were stained with Haematoxylin-Eosin (H&E), and Masson’s Trichrome (MT). The histological slides were evaluated using light microscopy (Olympus BX51; Olympus, Tokyo, Japan). One rat was sacrificed on day 14 of the experiment, and for each sample, four different locations of the wound bed were analyzed for histological study. The rest of the animals (n = 4) were sacrificed on the 21st day of the experiment for histological/histomorphometric analysis. Accordingly, epithelialization, inflammation, fibroplasia, granulation tissue formation and collagen deposition have been assessed in different groups, comparatively.

In histomorphometric study, the epithelialization was assessed semi-quantitatively on 5 point scale: 0 (without new epithelialization), 1 (25%), 2 (50%), 3 (75%), and 4 (100%). For these parameters, results were validated by a comparative analysis of one independent observer blinded to the treatment groups.

### Statistical analysis

In order to study the statistically significant difference among the fabricated scaffolds in the *in-vitro* experiments, one-way ANOVA (ANalysis Of VAriance) was adopted, followed by Tukey’s HSD (Honestly Significant Difference) post hoc test. Furthermore, all of the histological/ histomorphometric results were compared using Kruskal Wallis analysis. In the present study, statistical analyses were performed using the SPSS software, version 20.0 (SPSS, Inc., Chicago, USA). In the present study, the P-value of less than 0.05 was considered as the statistically significant difference between the results.

## Results and discussion

The results are reported in two parts, including different hydrogel substrates and the hybrid microsphere/hydrogel system. The optimal hydrogel structure was initially identified based on the results of the first section. Then, it was utilized as the substrate for fabricating the hybrid scaffold, the results of which are presented in the second section.

### Hydrogel substrates *in-vitro* experiments

#### Morphology

**Figure 1:**
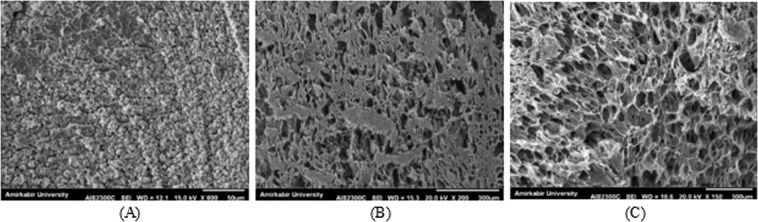
SEM images of PVA/SA hydrogel microstructures at different proportions of: (**A**) 90:10 (scale bar: 50 μm); (**B**) 70:30 (scale bar: 300 μm); (**C**) 50:50 (scale bar: 300 μm).

In the field of tissue engineering, microstructural properties of the scaffolds affect cell infiltration, proliferation, and function significantly^49^. Accordingly, Fig. 1 presents the SEM images of the cross-section morphology of hydrogel structures. As shown, many irregular aggregates were observed on the cross-section of the specimen with 10% SA concentration, which might be attributed to PVA crystallization^50^. In order to observe the described microstructure of this sample precisely, its SEM image is reported in higher magnification in Fig. 1. The non-porous structure of this sample was also due to the shrinkage of the polymer network during freeze-thawing and freeze-drying processes because of the influence of highly cross-linked PVA. Increasing the SA content decreases the shrinkage of the freeze-dried scaffolds because SA, as the second polymer in the structure of the samples, does not crosslink. No shrinkage was observed for the specimen with 50% SA, which resulted in uniform distribution of the pores inside the hydrogel structure, unlike the other two samples. Moreover, the average pore size and the number of pores in the scaffolds was increased with the increment of SA. These morphological results are compatible with the reported results in ref. ^33^.

In addition, the specimen containing 30% SA resulted in a low porous structure with the porosity percentage equal to 37%, wherein the heterogeneous distribution of the pores was quite evident. In contrast, increasing the SA proportion to 50% resulted in a highly porous structure with the porosity percentage of 52%, and the average pore size of 62 ± 16 μm, which belongs to the optimum range of 20–125 μm required for an efficient dermal fibroblast cell intrusion^51^. The mentioned quantified evaluations of the SEM images, including measuring the pore size and porosity, were calculated using ImageJ® software.

#### Swelling ratio

**Figure 2:**
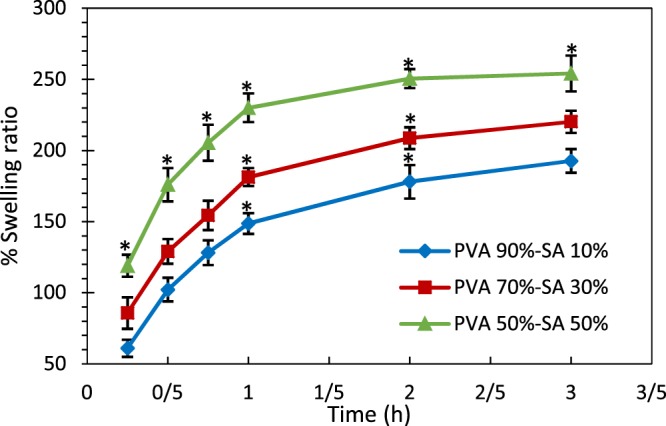
Swelling behavior of hydrogel structures in PBS. The * indicates p < 0.05 between the samples.

The results of the swelling test of the hydrogel structures for 3 h are illustrated in Fig. 2. According to the results, the variation in swelling ratio of all the specimens from the 2nd to the 3rd hour was less than 10%, which means that the swelling ratio of the samples reached equilibrium in 3 hours. As shown, at each time interval of the experiment, the higher the SA content of the scaffold, the higher the swelling ratio of the sample was. The statistical analysis of the results indicates that at each time interval, the specimens containing 50% SA, absorb a significantly higher amount of PBS in comparison with the other two samples (p < 0.05). This phenomenon was probably because the crosslinking did not occur between the SA chains, in addition to the SA high solubility in water, which resulted in the high swelling ability of SA. This finding can also be ascribed to the fact that the porosity percentage of the samples was increased by adding the SA content. Indeed, the increment of SA led to the formation of a more hydrophilic structure with a low degree cross-linking, which shows higher water uptake ability. Based on the results of ref. ^52^, highly cross-linked structures cannot retain much water inside their structure. It was also observed that the water absorption rate of all hydrogels decreased with time. These results are well in accordance with the obtained results in ref. ^30,33^.

#### Degradation rate

**Figure 3:**
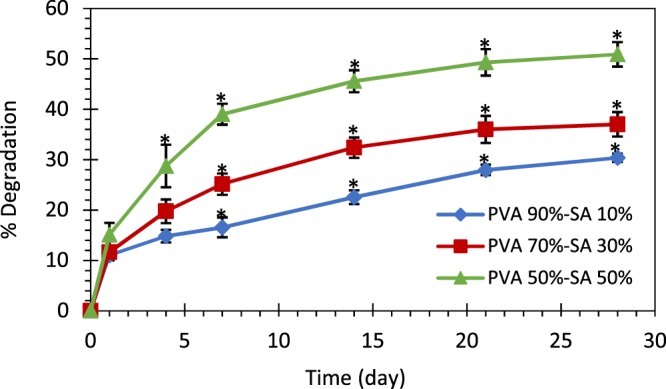
The degradation rate of different hydrogels. The * indicates p < 0.05 between the samples.

The optimized skin scaffold should degrade within 3 to 4 weeks at the wound site, depending on the wound type and severity. Accordingly, Fig. 3 indicates the degradation rate of the fabricated hydrogel substrates in 4 weeks. As observed, increasing SA concentration accelerated scaffold degradation in a way that the degradation rate of the samples with 50% SA was significantly higher than the other two specimens at each time interval (p < 0.05), except for day1. Such a difference was also observed between the two other samples from day 7 of the experiment (p < 0.05). This phenomenon was due to the cleavage of the polymer chain entanglements in the hydrogel structure because of the mitigation of cross-linking density by increasing the SA content, as reported in ref. ^33,53^. The high solubility of SA in water could also result in the scaffolds’ weight loss. Because most of the blended materials that did not crosslink and were only entrapped within the gel structure degraded in the early times, the slope of the degradation curves of all the samples decreased over time.

According to the results, all of the created scaffolds provide enough time for skin tissue regeneration, since none of them represented extensive degradation in 4 weeks. However, one should notice that the prolonged degradation of the scaffold can delay the wound healing process.

#### Mechanical properties

**Figure 4:**
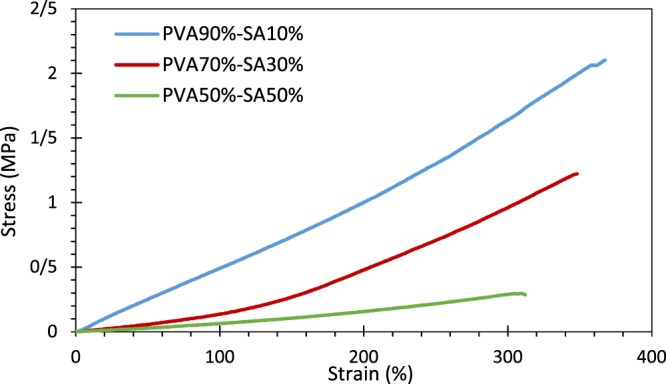
Stress-strain diagram of the hydrogel structures using the extension rate of 2.5 mm/min.

Adequate mechanical properties of a scaffold are crucial in skin tissue regeneration, affecting the differentiation fate of stem cells^54^. In this regard, the results of tensile testing of the fabricated hydrogel substrates are shown in Fig. 4 (only for one test of each sample) and Table 1. Based on this experiment, the maximum strength of the samples decreased with the increment of the SA portion (p < 0.05 between all three specimens). It was due to the reduced cross-linking density in the samples with higher SA percentage, as mentioned in ref. ^43^. However, no significant difference in elongation at break was observed between different hydrogels (p > 0.05). Furthermore, Young’s moduli of 0.59 ± 0.06, 0.16 ± 0.05, and 0.06 ± 0.02 MPa were achieved for the hydrogels with SA proportions of 10%, 30%, and 50%, respectively. This result suggested that Young’s modulus of the samples containing 10% SA was significantly higher than the two other specimens, resulting in a stiffer hydrogel (p < 0.05). These observations are compatible with the results in ref. ^30^. Accordingly, the hydrogel with 10% SA did not present the gel-like behavior and was highly stiff due to the strongly cross-linked PVA.

**Table 1:** The mechanical properties of the hydrogel structures.

	Elongation (%)	Tensile Strength (MPa)	Young’s modulus (MPa)
PVA 90%-SA 10%	394.000 ± 24.457	1.902 ± 0.141	0.589 ± 0.056
PVA 70%-SA 30%	331.425 ± 16.225	1.166 ± 0.056	0.160 ± 0.046
PVA 50%-SA 50%	330.827 ± 22.766	0.281 ± 0.033	0.057 ± 0.023
Hybrid System	425.900 ± 94.800	0.148 ± 0.072	0.023 ± 0.005

#### Water vapor transmission rate

**Figure 5:**
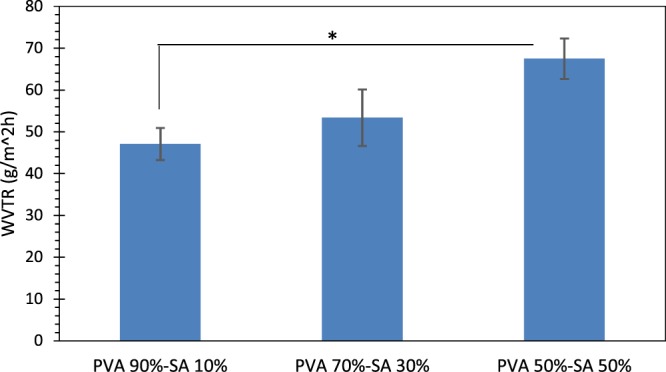
Water vapor transmission rate (WVTR) of the hydrogel samples. The * indicates p < 0.05 between the samples.

The ability of the skin scaffolds to reduce body liquid loss is an essential factor for improving the wound healing process, especially in burn victims^43^. Therefore, WVTR is measured in the fabricated scaffolds in the present study. According to the results (Fig. 5), the WVTRs of the samples containing 10%, 30%, and 50% SA were 47.08 ± 3.84, 53.36 ± 6.75, and 67.48 ± 4.84 g/m^2^h, respectively. The statistical analyses revealed that WVTR of the specimens with 50% SA was significantly higher than the ones with 10% SA (p < 0.05). Based on the data reported in ref. ^43^, the WVTR of some commercialized wound dressings vary from 33 (Op site) to 208 (Omiderm) g/m^2^h, which indicates that the WVTRs of the created scaffolds in the present study are in a suitable range for wound healing. Based on ref. ^43^, higher values of WVTR result in drying the wound area and the lower values of WVTR lead to wound exudates accumulation and a higher risk of bacterial growth.

#### Identification of the optimized hydrogel substrate

Based on the comparative results obtained from the hydrogel substrates evaluations, we selected the sample containing 50% PVA and 50% SA, as the optimized scaffold to prepare the hybrid system. The highly porous structure of this hydrogel with suitable pore size and homogenous pore distribution would be more efficient for cell penetration and proliferation. In addition, according to the WVTR and water absorption experiments, this scaffold is able to provide an appropriate moist environment for the wound, which improves its healing process.

Moreover, this hydrogel can be degraded with an appropriate rate, which is neither very slow, leading to a delay in wound healing nor very quick, which causes extensive degradation before tissue regeneration. The tensile testing showed relatively low strength and Young’s modulus for the hydrogel with 50% SA compared with the other two samples. To be more specific, this specimen exhibited more flexibility, as well as gel-like behavior required for an ideal skin scaffold, in contrast to the stiff structure of the sample with 10% SA.

Consequently, the hydrogel substrate of the hybrid microsphere/hydrogel system was fabricated using 50% SA and 50% PVA.

### Hybrid microsphere/hydrogel system *in-vitro* experiments

#### Morphology

**Figure 6:**
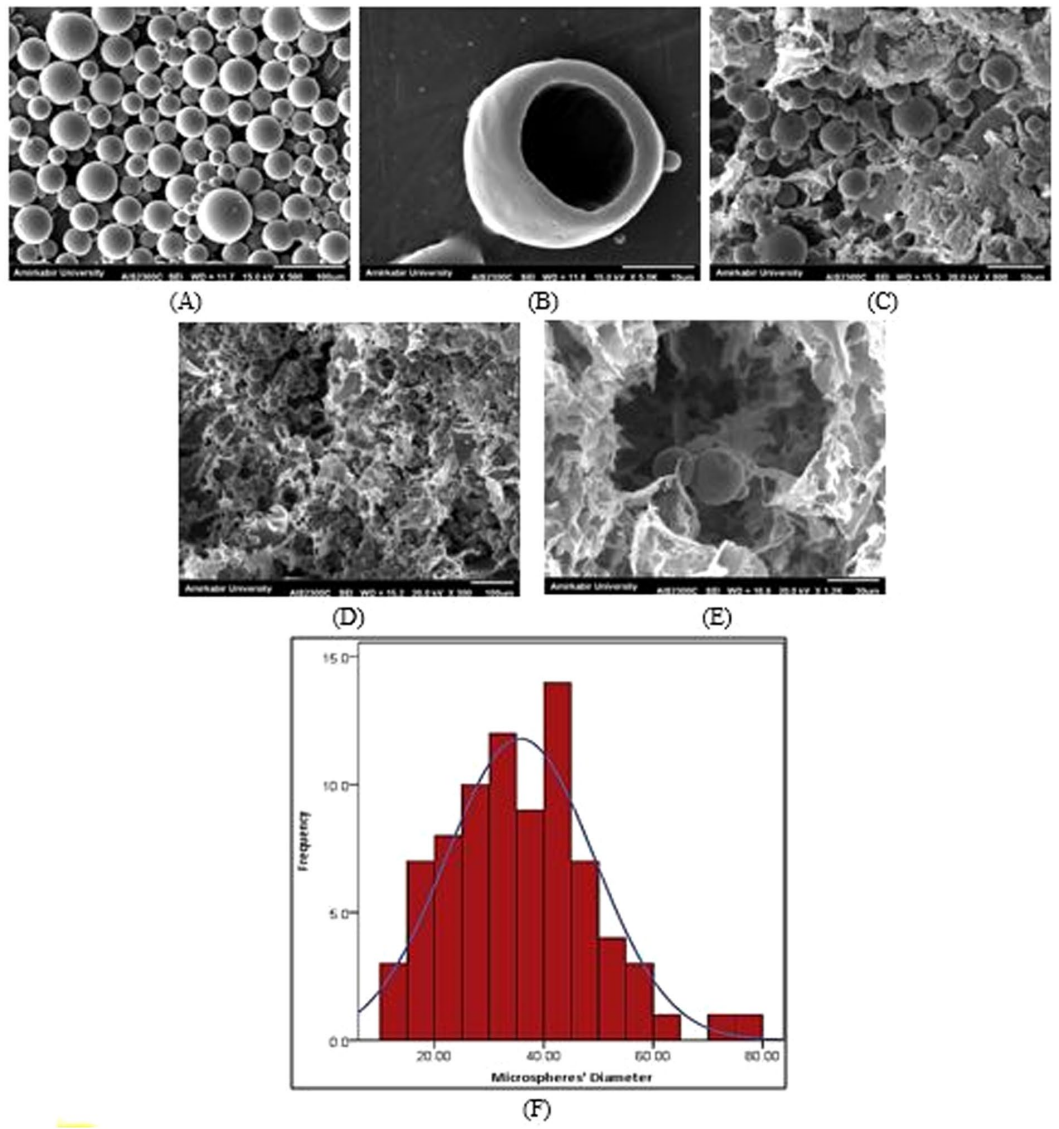
The SEM images of (**A**) PCL microspheres (scale bar: 100 μm), (**B**) fractured cross-section indicating the hollow space inside the PCL microsphere (scale bar:10 μm), (**C-E**) microstructure of hybrid system composed of PCL microspheres incorporated into the PVA/SA hydrogel (scale bars: 50, 100, and 30 μm, respectively), and (**F**) the histogram of the microspheres’ diameter.

The SEM images taken from PCL microspheres and the hybrid microsphere/hydrogel system are provided in Fig. 6. As shown in this figure, the microparticles appeared entirely spherical with a highly smooth and non-porous surface. The diameter of 80 microspheres was measured using ImageJ software, indicating their diameter equal to 35.67 ± 13.44 μm. Moreover, the successful incorporation of the microspheres into the optimized hydrogel substrate is evident in Fig. 6. The SEM images illustrated that the PCL microspheres were distributed inside the porous microstructure of the hydrogel while retaining their structure and spherical shape.

#### BSA encapsulation efficiency

The encapsulation efficiency (EE) value of BSA for PCL microspheres, which were created by the double-emulsion solvent evaporation technique was achieved equal to 60.8 ± 3.7%. This finding is consistent with the published results in refs. ^22,34^, in which the authors have reported the EE values of 62.4% and 62.9% for similar microspheres, respectively. They have also revealed that the concentration of the polymers utilized in the inner aqueous phase (PVA1%-PCL3%) has led to this high BSA entrapment efficiency. The higher EE is desirable since for the higher EE, less waste of the drug happens in the process of microspheres fabrication, which is essential, especially for expensive drugs such as bFGF.

#### *In-vitro* drug release

**Figure 7:**
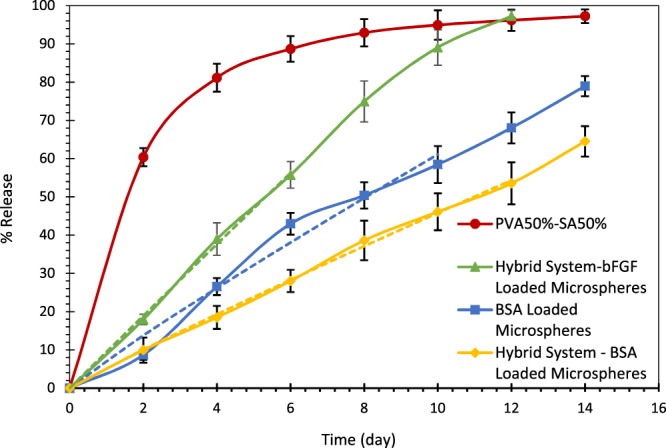
The release profile of BSA from PCL microspheres (blue curve with square markers), hydrogel structure (red curve with circular markers), microspheres embedded in the hydrogel substrate (yellow curve with rhombic markers), and bFGF release from the hybrid system (green curve with triangular markers). The dashed lines represent the curve fitting of the drug release profiles with the Peppas-Ritger equation, whose results are shown in Table 2.

In the first step, to determine the effect of the hydrogel substrate on the drug release profile of PCL microspheres, BSA release, as the model drug, was evaluated from the hybrid microsphere/hydrogel scaffold and microspheres alone. Direct drug release from the hydrogel as a common drug delivery approach in tissue engineering, was also assessed. In this regard, Fig. 7 shows the BSA release profile of the afore-mentioned systems for 14 days. The release profile of BSA from the hydrogel porous microstructure indicated a rapid release in the first 2 days (almost 60% of the BSA content of the samples released during this time), which is known as the burst release^55^. This phenomenon is not desirable, for the burst release of bFGF, as the target drug in the present study, may result in serious side effects due to the uncontrolled cell migration and proliferation. This high initial burst release could be attributed to the rapid diffusion of BSA molecules through the pores of the scaffold. The release of BSA molecules loaded close to the hydrogel surface also increased the initial release rate.

The BSA release profile from PCL microspheres is also shown in Fig. 7. According to the results, almost 79% of the encapsulated BSA content was released within 14 days. Fitting the release profile of BSA release from microspheres with the Peppas-Ritger equation resulted in n = 0.927, as indicated in Table 2. Based on ref. ^41^, n = 1 in the Peppas-Ritger equation shows zero-order drug release. Therefore, the BSA release from the microspheres is highly close to a zero-order and burst-free release behavior. Furthermore, embedding the microspheres into the hydrogel substrate did not affect their burst-free zero-order release behavior. As shown in Table 2, fitting the BSA release curve from the hybrid system with the Peppas-Ritger equation gave n = 0.943, which is highly close to n = 1 and zero-order release. Besides, using the scaffold as a career for the microspheres slowed down the BSA release rate due to the presence of the hydrogel substrate, resulting in a more sustained drug release in the hybrid microsphere/hydrogel system. This finding can be ascribed to a consecutive release mechanism, wherein the drug molecules diffuse initially through the microspheres and subsequently through the hydrogel structure. As indicated in Fig. 7, the percentage of the released drug reached 64.5% for the hybrid system in 14 days, still providing enough time for drug delivery to the wound site.

**Table 2:** The results of fitting the drug release profiles with the Peppas-Ritger equation.

	k	n	R^2^
BSA Loaded Microspheres	7.233	0.927	0.990
Hybrid System – BSA Loaded Microspheres	5.225	0.943	0.999
Hybrid System – bFGF Loaded Microspheres	9.503	0.993	0.999

According to the results, the prepared hybrid system can be used for prolonged drug delivery purposes, which reduces the utilized amount of the drug and its side effects. Thus, the release mechanism required for bFGF in this study was provided through the proposed hybrid system, which resulted in a sustained, controlled release of bFGF during the wound healing process. Similarly, ref. ^24,26,27^ have reported that the encapsulation of GFs in microspheres embedded in hydrogel structures results in a remarkable control on the drug release rate, compared with their release from hydrogels or microspheres alone.

Moreover, the release profile of bFGF from PCL microspheres incorporated into the hydrogel substrate is also shown in Fig. 7. As observed, the release profile of bFGF confirms the sustained and controlled release mechanism of growth factors through the hybrid system (fitting the bFGF release profile with the Peppas-Ritger equation gave n = 0.993, which approves the zero-order release mechanism of bFGF). In addition, although the burst-free and zero-order release profile of bFGF was achieved, its release is more rapid than BSA. This finding could be ascribed to the less molecular weight of bFGF (17.9 kDa) in comparison with BSA (66.5 kDa) because the time required for drug diffusion through a polymer structure is proportional to the square root of the molecular weight, based on Graham’s law on diffusion^56^.

#### Degradation rate

**Figure 8:**
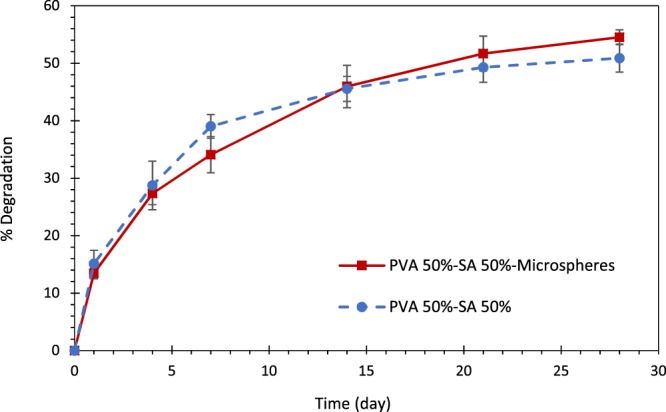
The degradation rate of the hybrid system (solid line) compared with its hydrogel substrate (dashed line). No significant difference observed between the two samples at each time point (p > 0.05).

The degradation rate of the prepared hybrid system was assessed prior to the *in-vivo* study, considering the importance of scaffold degradation mechanism in tissue regeneration. Accordingly, Fig. 8 presents the degradation rate of the hybrid scaffold in comparison with the optimized hydrogel substrate, which discussed previously in 4 weeks. As shown, there was no significant difference between the two diagrams (p > 0.05). Hence, the hybrid system provides the desired degradation rate for the wound healing process, similar to the optimized hydrogel structure.

#### Mechanical properties

**Figure 9:**
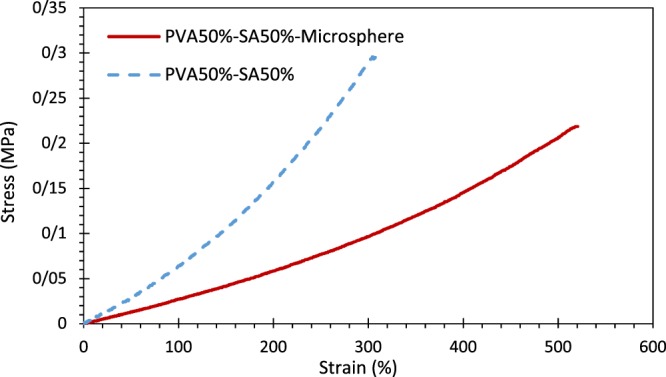
Stress-strain diagram of the hybrid system (solid red line) in comparison with its hydrogel substrate (dashed blue line) using the extension rate of 2.5 mm/min.

The results of tensile testing of the hybrid scaffold are shown in Fig. 9 (only for one test of each sample) and Table 2. As observed, the maximum tensile strength measured for the hybrid system (0.148 ± 0.072 MPa) was less than the optimized hydrogel substrate (0.281 ± 0.033 MPa). It could be attributed to the incorporation of microspheres into the hydrogel structure, which leads to the decrement of polymer chain entanglements through the rupturing of the polymer network, as mentioned in ref. ^57^. However, statistical analyses revealed that the mentioned difference was not significant (p > 0.05). Besides, the elongation at break increased in the hybrid system, but not significantly (p > 0.05). Furthermore, the hybrid scaffold exhibited more flexibility in comparison with the hydrogel without microspheres (Young’s modulus was equal to 0.057 ± 0.023 MPa for the hydrogel substrate, whereas, it was equal to 0.023 ± 0.005 MPa for the hybrid system. Based on the results, the developed skin scaffold possesses sufficient mechanical properties for the clinical application as the wound dressing.

#### Antibacterial characteristics

**Figure 10:**
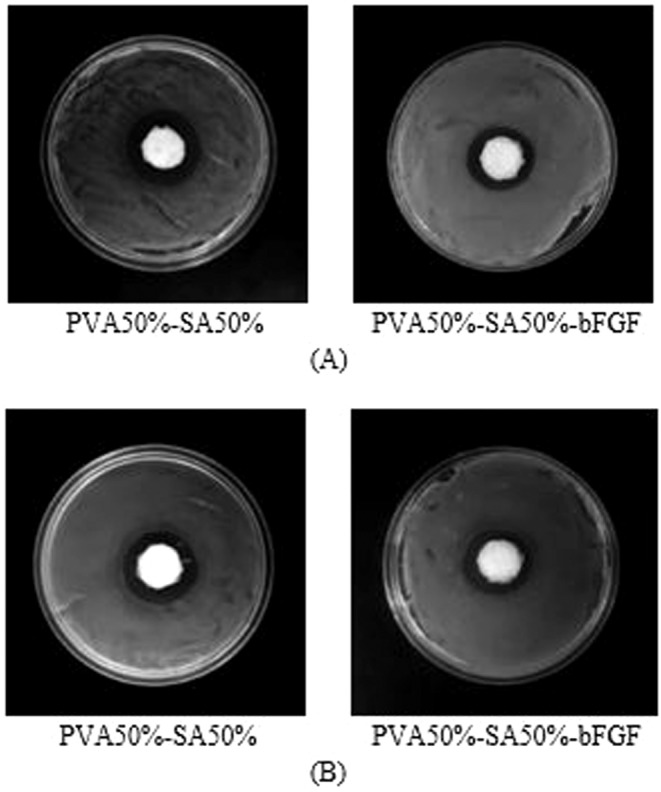
Antibacterial evaluation of the drug-loaded hybrid system compared with the blank hydrogel against (**A**) Staphylococcus aureus (S. aureus), and (**B**) Escherichia coli (E. coli). The diameter of all samples is 17 mm.

The antibacterial activity is one of the critical requirements of skin scaffolds to prevent bacterial infection as an underlying cause of postponement in the wound healing process^57,58^. In this regard, Fig. 10 illustrates the antibacterial activity of the hybrid bFGF-loaded microsphere/hydrogel system compared with the hydrogel alone as the control sample. Additionally, Table 3 presents the measured bacterial inhibition zone corresponding to these scaffolds. As observed, the optimized hydrogel, with or without the bFGF-loaded microspheres, showed antibacterial activity against both bacterial species of S. aureus and E. coli. According to the results, the fabricated scaffolds are able to combat the common pathogens associated with acute wounds. Furthermore, the statistical analyses revealed that the incorporation of bFGF-loaded microspheres into the hydrogel substrate did not change the inhibition zone significantly (p > 0.05). Similarly, the antibacterial activity of pure PVA was investigated against S. aureus, whose result is shown in Fig. S2 in the Appendix. Based on the result, pure PVA did not show any antibacterial activity against the mentioned bacteria.

**Table 3:** Bacterial inhibition zone of the different scaffolds.

Sample name	Inhibition zone (mm)	
	S. aureus	E. coli
PVA50%-SA50%	7.7 ± 1	7.2 ± 0.7
PVA50%-SA50%-bFGF	5.8 ± 0.7	6.1 ± 1.1

#### MTT cell viability assay

**Figure 11:**
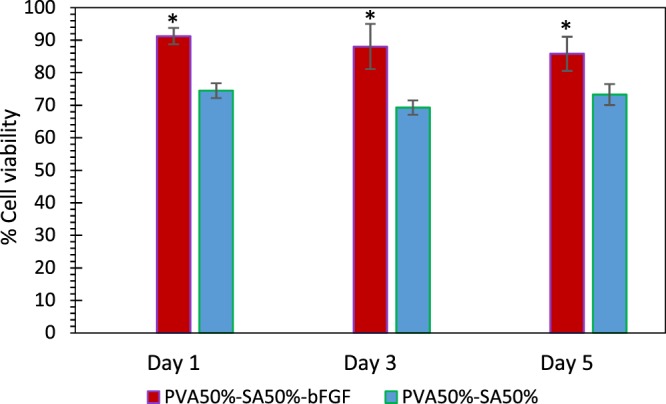
L929 cells proliferation on days 1, 3, and 5 for different scaffold formulations, compared with TCP as representing 100% cell viability. The * indicates p < 0.05 between the two different samples at each time point.

Cell adhesion and proliferation are other crucial factors in designing skin scaffolds so that they can support and guide tissue regeneration^48,59^. In this regard, Fig. 11 displays the results of the cell-matrix interactions using MTT cell viability assay in 5 days. As observed, the scaffolds did not present any cytotoxic effect, for materials can be labeled as non-cytotoxic when the cell viability remains above 70% (according to the guideline for determining the *in-vitro* cytotoxicity of medical devices (DIN EN ISO 10993-5))^60,61^. However, the cell viability percentage of the blank hydrogel was equal to 69.3% on day 3, even though PVA and SA, as well as their degraded particles, are well-known as biocompatible materials. This outcome might be due to the hydrogel degradation over time and removal of the degraded material and their attached cells while replacing the culture medium on day 2. Nevertheless, the degraded material and adhered cells remain in the wound site at *in-vivo* conditions. In addition, the cells were able to proliferate within all structures, confirming cell adhesion.

Furthermore, the statistical analyses revealed that bFGF-loaded PCL microspheres promoted the proliferation of L929 cells in comparison to the hydrogel alone (p < 0.05 at each time point of the experiment between the two different groups). It can be due to the stimulating effect of bFGF on fibroblast cell migration and proliferation^12,62^, which was continuously observed in 5 days of the test, due to the sustained and controlled release of protein from PCL microspheres. However, the cell viability percentage did not change significantly over time for any of the evaluated scaffolds (p > 0.05).

#### Cell attachment analysis

**Figure 12:**
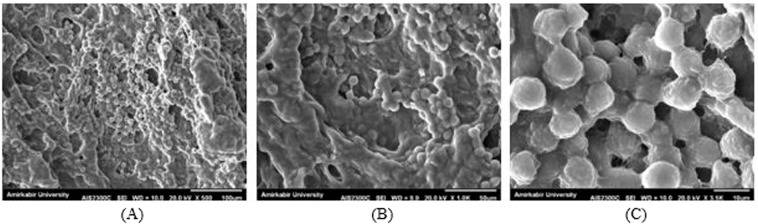
The SEM images of attached L929 cells (**A**) ×500 magnification (scale bar: 100 μm), (**B**) ×1.0 k magnification (scale bar: 50 μm). (**C**) Passively adhered L929 cells to the hybrid microspheres/hydrogel system on the 5th day after cell seeding (×3.5 k magnification, scale bar: 10 μm).

Figure 12 illustrates the morphology of L929 fibroblasts seeded on the hybrid microsphere/hydrogel system after 5 days. These SEM images indicate that the cells on the scaffold surface were able to adhere well, but many of them could not spread on the scaffold surface after attachment. This type of cell-polymer interaction is interpreted as passive adhesion^63^; wherein cells retain their spherical shape, as observed in Fig. 12B. In this type of attachment, cells initiate to adhere on the hydrogel substrate by a combination of complex physicochemical interactions between the cell membrane and the polymer surface^64^. However, active adhesion, leading to integrin binding, does not adequately occur. As a result, L929 cells did not spread and became flattened in the present study. Similar results have been reported for electrospun PVA/SA/nHAP fiber membranes^65^, PVA membranes^66^, and silk sericin/PVA scaffolds^67^.

Although PVA/SA hydrogel was not cytotoxic towards fibroblasts based on the results of MTT cell viability assay in Fig. 11, this scaffold inhibited cell proliferation to some extent. ref. ^68^. has made a similar claim for the non-spreading L929 cells on the chitosan membrane surface. Furthermore, the presence of bFGF, as a biological molecule within the proposed hydrogel structure, provides a chemical cue to the cells that can control their behavior^63^.

### *In-vivo* assays

According to the results obtained from *in-vitro* experiments, the hybrid “bFGF-encapsulated microsphere/hydrogel” system (H-bFGF) and drug-free optimized hydrogel structure (O-H) were selected for the *in-vivo* assessments.

#### Wound closure

**Figure 13:**
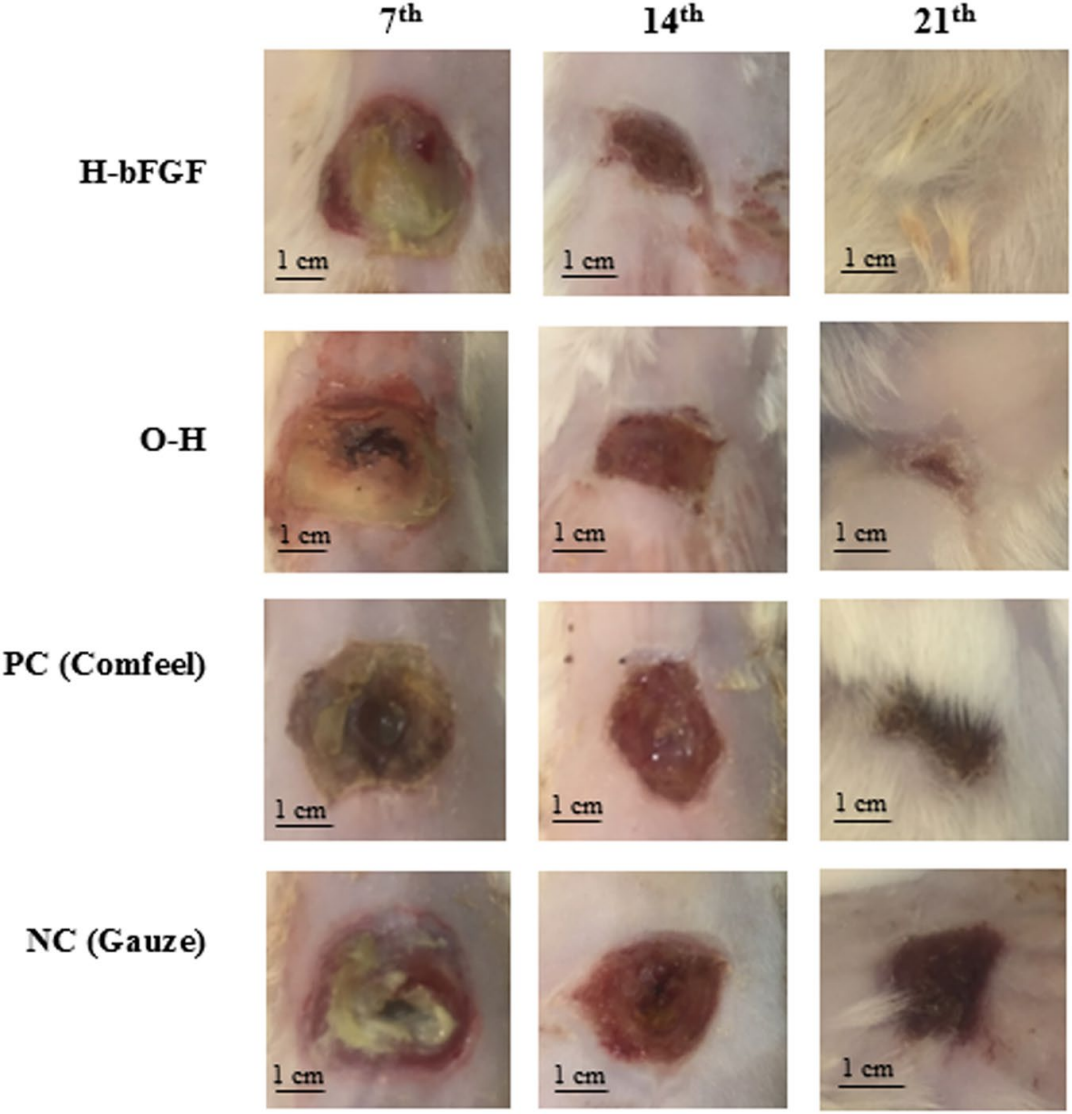
Images of burn-wound sites of the different treatment groups on the 7th, 14th, and 21st days after grafting.

**Figure 14:**
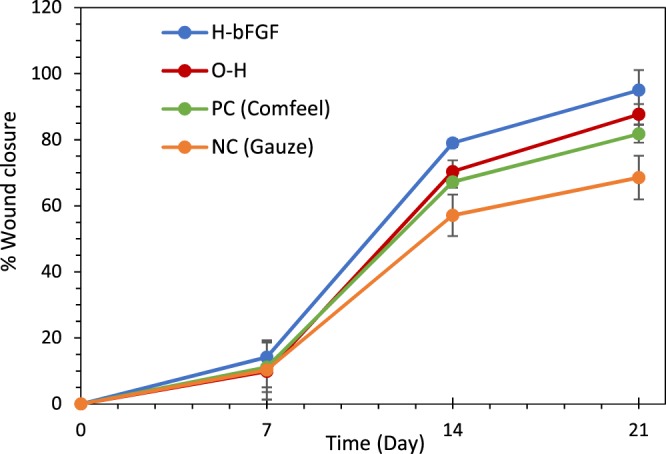
The wound closure percentage for various treatment groups in 21 days.

The images of the burn-wound healing process in different groups on the 7th, 14th, and 21st days of the *in-vivo* experiment are demonstrated in Fig. 13. The detailed quantitative results of the wound closure percentages for the mentioned groups are presented in Fig. 14, as well. According to the results, the wound size varied considerably in all of the examined cases in 21 days of the assessment (p < 0.05 for all of the samples, except for the NC group between the 14th and 21st days of the treatment). The noticeable healing effect of the present hybrid system and the hydrogel substrate without drug-loaded microspheres on the burn-wound was also evident as compared to gauze and Comfeel, as the control groups. Based on the results, although no significant change was observed between different groups on the 7th day of the test, the area of the wounds treated with H-bFGF and O-H was significantly smaller than the ones treated with NC on 14th and 21st days of the experiment (p < 0.05). Moreover, the wound area of the group with H-bFGF was significantly smaller than the one in the NC group on day 14 (p < 0.05). Accordingly, the wound contraction percentage for the H-bFGF group reached above 79% on the 14th day and was also about 95% on the 21st day. This finding could be attributed to the bioactivity of bFGF inside the H-bFGF scaffold, which led to the promotion of epithelialization and collagen synthesis and consequently enhanced efficient tissue regeneration^13^. Besides, it might be due to the bFGF’s ability to develop a vascular structure by signal sending to a distinguished group of cells^69^. In the case of the O-H group, the approximate wound closure percentage of 88% was attained on day 21.

#### Histological/histomorphometric analysis

**Figure 15:**
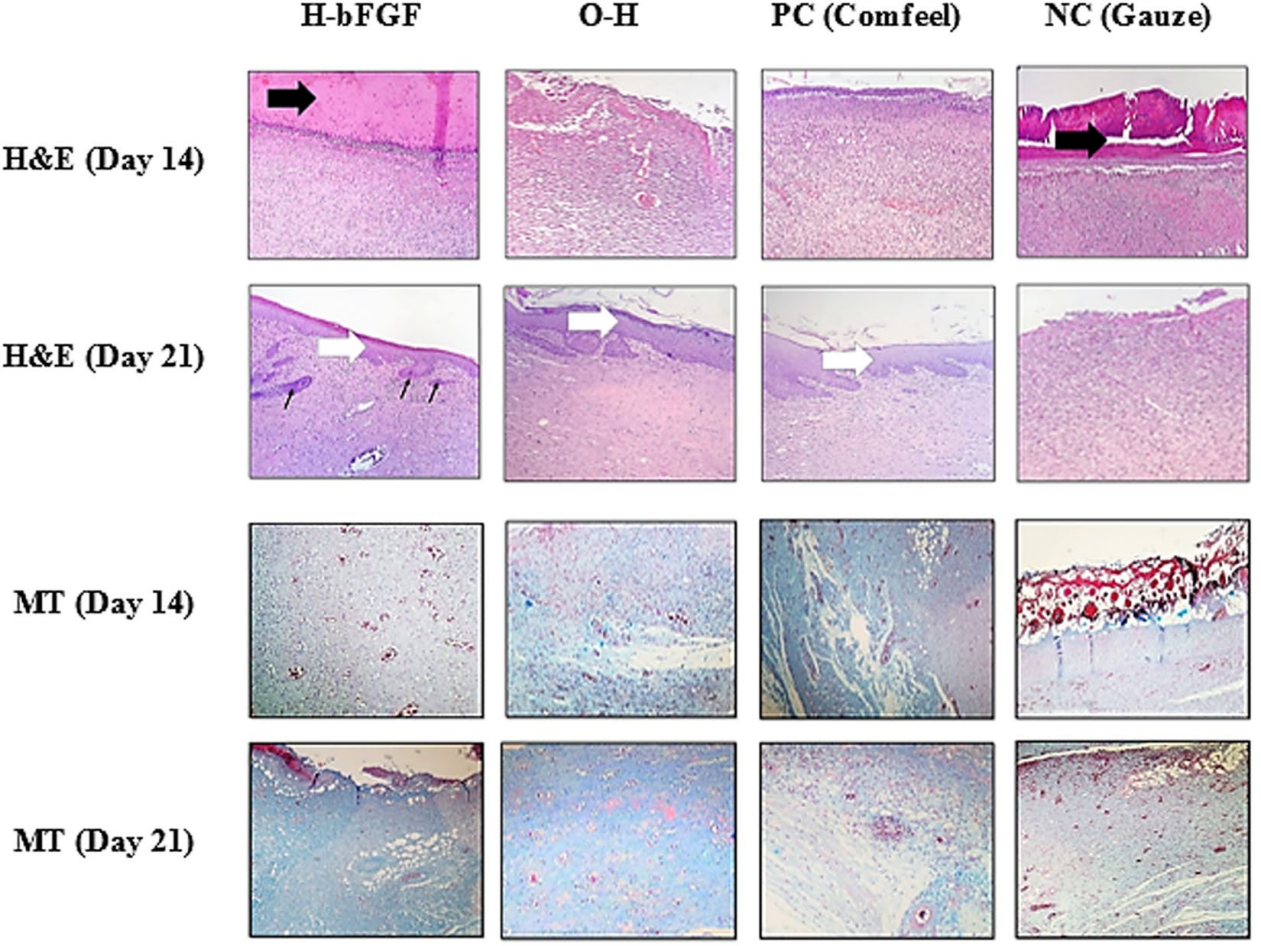
Representative histological sections of skin tissue regeneration at 14 and 21 days post-treatment. The sections were stained with MT and H&E (black and white arrows point to crusty scab and epithelial layer, respectively. Thin arrows indicate the rejuvenation of hair follicles) (×400 magnification).

Histological analyses of the burn-wounds at 14th and 21st days of the healing process, performed by H&E and MT staining on harvested tissue samples, are illustrated in Fig. 15. Polymorphonuclear inflammatory cells (PMNs) infiltration and granulation tissue formation were observed during the histopathological evaluation of the NC group at 14 and 21 days of the assessment. However, the epidermal layer was not formed and a crusty scab had covered the wound. According to the histomorphometric results indicated in Table 4, amongst all experimental groups, re-epithelialization in NC one was minimum and immature granulation tissue mostly filled its wound bed (P < 0.01). For the group treated with the commercialized Comfeel wound dressing (PC), there was a close similarity to the NC group on day 14 (Fig. 15); the wound area was covered with a crusty scab, and inflammation was evident in the wound bed, without epidermal outgrowth. On the 21st day, although the number of inflammatory cells significantly decreased compared to the NC group at the same time, a narrow layer of epithelial cells had formed.

**Table 4:** Histomorphometric analysis of different Table. 5. The progress of collagen synthesis of various experimental groups at 14 and 21 days post-treatment. treatment groups on days 14 and 21 after grafting.

Group	Epitheliogenesis score (N = 4)
H-bFGF	1,0,1,2 (14 d)* 4,4,4,4 (21 d)***
O-H	1,1,0,0 (14 d)* 3,3,3,4 (21 d)**
PC (Comfeel)	0,0,1,1 (14 d)* 3,3,2,2 (21 d)**
NC (Gauze)	0,0,0,0 (14 d) 0,0,0,1 (21 d)
**Group**	**Collagen density (%) (N** = **4)**
H-bFGF	38.7 ± 3.7**(14) 74.7 ± 4.1***(21)
O-H	35.7 ± 4.2*(14 d) 71.7 ± 4.5***(21 d)
PC (Comfeel)	32.5 ± 4.5*(14 d) 44.2 ± 4.3**(21 d)
NC (Gauze)	25 ± 2.9 (14 d) 30.2 ± 3.3 (21 d)

*, **, ***values indicate treatment group versus un-treatment group (control); *P < 0.05, **P < 0.01, ***P < 0.001.

Micrograph corresponding to the O-H group at 14 and 21 days after grafting revealed an inflammation relatively similar to PC. The epidermal layer also had not been created in the wound bed until the 14th day. During the third week, re-epithelialization progressed quickly, as led to positive findings in the following treatment; the epidermis and dermis initiated to form and complete coverage of new epithelium with the existence of rete ridges was observed on the 21st day for the O-H group, as an indicator of accelerated tissue regeneration. Furthermore, histopathological evaluation of H-bFGF illustrated a noticeable inflammation reduction at day 14 compared to the others. This group showed more resemblance to the healthy skin at day 21, with thin epidermis and normal rete ridges, rejuvenation of skin appendages (hair follicles) and normal thickness of skin layers, demonstrating the best cosmetic appearance. All these judgments regarding the epithelialization process were in good agreement with the histomorphometric analysis (Table 4), which confirmed the best re-epithelialization for wounded skin treated with H-bFGF. Besides, the O-H treatment group obtained more epitheliogenesis score compared to PC.

The improvement in collagen synthesis during the formation of granulation tissue and matrix remodeling, assessed with MT staining, is shown in Table. 5. Collagen fibers were stained blue-green via this method, in which the intensity of this color correlates with the relative amount of deposited total collagen and indicates the progress of collagen synthesis and tissue remodeling. H-bFGF and O-H groups were found to have the greatest collagen density, respectively, compared to the control groups at both 14th and 21st days of healing. In contrast, the lowest rate belonged to NC one. Also, more mature collagen (on day 21) was detected for the group treated with H-bFGF to recreate the ECM and repair the skin tissue effectively.

Overall, H-bFGF treated group indicated more correspondence to normal skin compared to others, suggesting the positive influence of the developed scaffold on the wound healing process.

## Conclusions

In our previous studies, we developed different types of scaffolds for tissue regeneration^8,9,22,70^. In the current study, a novel type of skin scaffold capable of drug delivery, based on hybrid utilization of PCL microspheres embedded into the PVA/SA composite hydrogel, was introduced to accelerate cell-induced tissue regeneration. The appropriate blending of SA and PVA polymers used in the proposed hydrogel caused to develop an optimized porous structure, as an imitator of skin tissue ECM, which possessed the adequate mechanical properties, gel-like physics, sufficient degradation rate, high swelling ability and an appropriate WVTR. The addition of PCL microspheres encapsulating bFGF, as a key stimulator of fibroblast migration and proliferation, to the hydrogel substrate created a burst-free and sustained release kinetics of this GF. The fabricated scaffold inhibited S. aureus and E. coli growth as well. Furthermore, the hybrid scaffold showed no cytotoxic effect *in-vitro* regarding the MTT assay and the cells were able to proliferate within the structure. The *in-vivo* evaluations also indicated that the constructed hybrid scaffold effectively accelerated the burn-wound healing process through promoting epithelialization and collagen deposition.

## Supplementary information

Appendix.

## Data Availability

The data in this study is available upon request.
