# Supplementary material for: Development of a polyvinyl alcohol/sodium alginate hydrogel-based scaffold incorporating bFGF-encapsulated microspheres for accelerated wound healing
Source: Sci Rep. 2020 Apr 30;10:7342. doi: 10.1038/s41598-020-64480-9 (PMC7193649; doi:10.1038/s41598-020-64480-9)
Supplement: Supplementary file 1 — Appendix. [file 41598_2020_64480_MOESM1_ESM.pdf]

Development of a polyvinyl alcohol/sodium alginate hydrogel-based scaffold incorporating bFGF-encapsulated microspheres for accelerated wound healing

Maedeh Bahadoran[a], Amir Shamloo[a], Yeganeh Dorri Nokoorani[a]
[a] Department of Mechanical Engineering, Sharif University of Technology, Tehran, Iran

Appendix

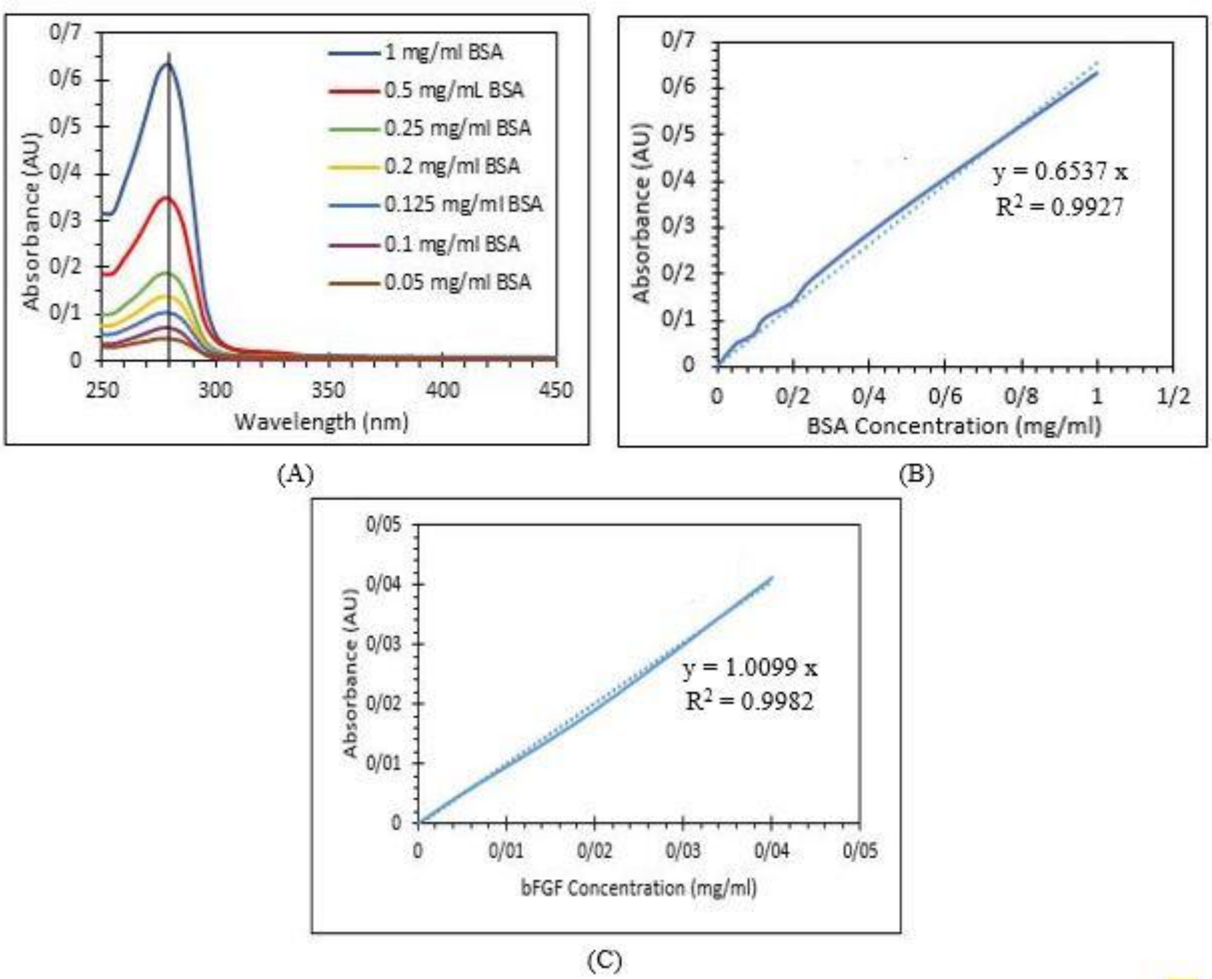

Fig.S1. (A) Absorption spectra of BSA at different concentrations; (B) Calibration curve for BSA at 280 nm; (c) Calibration curve for bFGF at 280 nm.

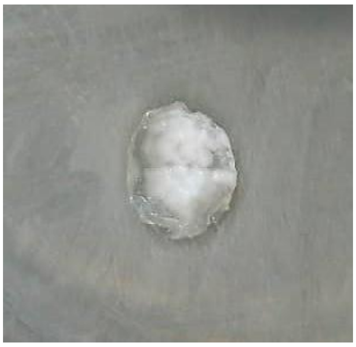

Fig.S2. The antibacterial activity of pure PVA sample against S. aureus. The experiment shows no antibacterial activity for this specimen against the mentioned bacteria.
